# Comparison of Nefopam-Based Patient-Controlled Analgesia with Opioid-Based Patient-Controlled Analgesia for Postoperative Pain Management in Immediate Breast Reconstruction Surgery: A Randomized Controlled Trial

**DOI:** 10.3390/jcm13123490

**Published:** 2024-06-14

**Authors:** Jaewon Huh, Noori Lee, Minju Kim, Hoon Choi, Deuk Young Oh, Jangyoun Choi, Wonjung Hwang

**Affiliations:** 1 Department of Anesthesiology and Pain Medicine, Seoul St. Mary’s Hospital, College of Medicine, The Catholic University of Korea, Seoul 06591, Republic of Korea; ether@catholic.ac.kr (J.H.); nurilee05@catholic.ac.kr (N.L.); minju1025@daum.net (M.K.); hoonie83@catholic.ac.kr (H.C.); 2 Department of Plastic and Reconstructive Surgery, Seoul St. Mary’s Hospital, College of Medicine, The Catholic University of Korea, Seoul 06591, Republic of Korea; ehyun@catholic.ac.kr (D.Y.O.); jycprs@gmail.com (J.C.)

**Keywords:** analgesia, patient-controlled, breast reconstruction, nefopam, opioids, postoperative pain

## Abstract

**Background/Objectives**: Immediate breast reconstruction surgery (BRS) often leads to significant postoperative pain, necessitating effective analgesia. This study aimed to compare the analgesic efficacy of patient-controlled analgesia (PCA) containing nefopam with that of PCA containing opioids alone in patients undergoing BRS. **Methods**: A prospective, double-blind, randomized controlled trial was conducted on 120 patients undergoing immediate BRS after mastectomy. Patients were randomly allocated to receive PCA with fentanyl alone (Group F: fentanyl 10 mcg/kg), fentanyl and nefopam (Group FN: fentanyl 5 mcg/kg + nefopam 1 mg/kg), or nefopam alone (Group N: nefopam 2 mg/kg). Pain intensity (expressed in VASr and VASm), opioid consumption, and opioid-related complications were assessed. **Results**: PCA with nefopam, either alone or in combination with opioids, demonstrated non-inferior analgesic efficacy compared to PCA with fentanyl alone. At 24 h postoperatively, the VASr scores were 2.9 ± 1.0 in Group F, 3.1 ± 1.2 in Group FN, and 2.8 ± 0.9 in Group N (*p* = 0.501). At the same timepoint, the VASm scores were 4.1 ± 1.2 in Group F, 4.5 ± 1.5 in Group FN, and 3.8 ± 1.4 in Group N (*p* = 0.129). Significant differences among the three groups were observed at all timepoints except for PACU in terms of the total opioid consumption (*p* < 0.0001). However, there were no significant differences in opioid-related complications among the three groups. **Conclusions**: PCA with nefopam, whether alone or in combination with opioids, offers non-inferior analgesic efficacy compared to PCA with fentanyl alone in patients undergoing immediate BRS.

## 1. Introduction

Immediate breast reconstruction surgery (BRS) following mastectomy often leads to significant postoperative pain [1]. Female patients tend to experience higher levels of pain intensity and a greater incidence of postoperative nausea and vomiting (PONV) [2], associated with a reduced quality of recovery and lower satisfaction levels postoperatively.

Traditionally, opioids have been the primary analgesic used during the perioperative period. However, common side-effects associated with opioid administration include sedation, nausea, vomiting, constipation, urinary retention, respiratory distress, opioid tolerance, and physical dependence. Additionally, there are concerns regarding the potential for persistent postoperative opioid use following acute exposure during this period. Consequently, healthcare providers are increasingly adopting opioid-sparing multimodal analgesia strategies for minimizing opioid use while ensuring effective postoperative pain management [3].

As part of these efforts, patient-controlled analgesia (PCA) incorporates various non-opioid agents to decrease opioid consumption. Nefopam is a non-opioid analgesic that acts centrally by inhibiting serotonin, norepinephrine, and dopamine reuptake and modulating glutaminergic transmission via N-methyl-D-aspartate receptors [4,5]. Previous studies have shown that PCA, including nefopam, either alone or combined with opioids, offers comparable postoperative pain relief with significantly reduced opioid consumption and associated complications compared to PCA with opioids alone [6,7,8,9,10]. However, there is a lack of studies focused specifically on nefopam’s analgesic efficacy as an adjuvant or primary agent in PCA for patients undergoing BRS. Since the severity of postoperative pain varies based on the type and extent of surgery, previous studies conducted on other surgical procedures do not necessarily guarantee the effectiveness of nefopam in BRS.

We hypothesized that PCA containing nefopam, either alone or in combination with opioids, could provide equally effective pain control compared to PCA containing opioids alone, while reducing opioid consumption and opioid-related complications after BRS. Therefore, this study compared the analgesic efficacy of PCA containing nefopam with that of PCA containing opioids alone in patients undergoing immediate BRS following mastectomy.

## 2. Materials and Methods

### 2.1. Study Population

This prospective, double-blind, randomized controlled trial was approved by the institutional review board and ethics committee (approval number: KC21MISI0819), and registered with the Clinical Research Information Service (KCT0006970). This study was conducted in accordance with the Declaration of Helsinki after obtaining informed consent from all participants.

This study enrolled 120 patients aged 20–75 years, with an American Society of Anesthesiologists physical status classification (ASA) of I–III, who were scheduled for elective immediate BRS following mastectomy under general anesthesia between January 2022 and July 2023. Exclusion criteria were a history of drug abuse, chronic pain, psychiatric or neurologic disorders, critical bradycardia or arrhythmia, severe cardiovascular disease, severe renal or hepatic disease, pregnancy or breast-feeding, allergy to the study drugs, previous PONV experience, and refusal to participate in the study. 

### 2.2. Randomization and Intervention

A total of 120 patients were randomly assigned to one of three PCA groups (F: fentanyl alone; FN: fentanyl and nefopam; N: nefopam alone). A computer tool was used for randomization. To ensure allocation concealment, intravenous PCA was prepared by an anesthesiologist who was not involved in the anesthesia management or outcome assessment of the patients. Since identity labels were only affixed to the PCA equipment, both patients and physicians who managed the patients or assessed the outcomes were blinded to group allocation.

Based on previous studies indicating that a 20 mg analgesic dose of nefopam is equivalent to 6–12 mg of morphine or 50 mg of meperidine [11,12], the following equivalent doses were established: nefopam 20 mg = morphine 10 mg = fentanyl 100 mcg. For each group, PCA was prepared by combining the following doses with normal saline to achieve a total volume of 100 mL:

Group F: fentanyl 10 mcg/kg

Group FN: fentanyl 5 mcg/kg + nefopam 1 mg/kg

Group N: nefopam 2 mg/kg

The PCA devices (AutoMed 3200^®^, ACE Medical Corp., Ltd., Seoul, Republic of Korea) were programmed to deliver a basal infusion rate of 1 mL/h, a bolus dose of 1 mL with a lockout time of 15 min, and a total allowable volume of 100 mL for all groups.

### 2.3. General Anesthesia and Postoperative Management

No premedication was administered before surgery. Patients underwent general anesthesia with standardized monitoring, including electrocardiography, non-invasive blood pressure monitoring, pulse oximetry, oxygen saturation, end-tidal carbon dioxide (EtCO_2_), and bispectral index (BIS). Anesthesia induction was achieved using propofol and remifentanil, with target concentrations of 3–5 mcg/mL and 2–4 ng/mL, respectively, via target-controlled infusion devices. Rocuronium (0.6 mg/kg) was administered for muscle relaxation followed by tracheal intubation. Subsequently, all patients received dual antiemetics, including 5 mg of dexamethasone and 75 mcg of palonosetron. Anesthesia was maintained using propofol (target concentration: 2.0–4.0 mcg/mL) and remifentanil (target concentration: 2.0–4.0 ng/mL) to ensure hemodynamic stability within 20% of the baseline values and BIS of 40–60. Mechanical ventilation parameters were set with FiO_2_ of 0.5, tidal volume of 6 mL/kg, and EtCO_2_ of 35–40 mmHg. Intravenous crystalloids were administered at a rate of 4 mL/kg/h through a warming device. Core body temperature was continuously monitored using an esophageal probe and maintained above 36 °C throughout the surgery. Forced-air warming blankets were applied as necessary. Intravenous acetaminophen (1 g) was administered to all patients 30 min before the end of surgery. At the end of surgery, sugammadex (2–4 mg/kg) was administered based on the TOF ratio, followed by extubation.

PCA was initiated for all patients upon arrival at the postoperative anesthetic care unit (PACU). Pain intensity was assessed using a visual analog scale (VAS), both at rest (VASr) and during movement (VASm). Pain scores were recorded at 10 min intervals in the recovery room and every 3 h on the ward. Additional analgesics were administered if the VAS score exceeded 4 despite PCA use. In the recovery room, fentanyl (50 mcg) was administered as supplemental analgesia, while on the ward, tramadol (50 mg) and pethidine (25 mg) were administered. Intravenous acetaminophen (1 g) was administered 12 h postoperatively as a regular dose.

Palonosetron (75 mcg) was pre-emptively administered on the day after surgery. In cases of PONV, intravenous metoclopramide (10 mg) was administered additionally. If PONV persisted despite antiemetic administration, intravenous PCA was discontinued.

### 2.4. Outcome Measurement

The primary outcome was the VAS score 24 h after the surgery. Secondary outcomes included VASr and VASm at 1, 6, 12, and 24 h postoperatively; cumulative opioid and PCA usage; and the occurrence of opioid-related complications. Cumulative opioid usage was derived from both PCA and rescue opioids at each timepoint, and converted to morphine equivalent dose (MED) for standardized comparison. Opioid-related complications included nausea/vomiting, dizziness, ileus, urinary retention, respiratory depression, and hypotension. Additionally, the need for rescue analgesics and the frequency of their administration were documented.

### 2.5. Statistical Analysis

We hypothesized that analgesia with nefopam monotherapy or fentanyl–nefopam polytherapy would be non-inferior to fentanyl monotherapy. The sample size was determined based on the primary outcome in accordance with the non-inferiority hypothesis. Considering a VAS score difference of <1 point as clinically insignificant, we set 1.0 as the non-inferiority margin (δ). Based on a previous study [10], the mean VAS score at 24 h after surgery was expected to be 4.20 for Group N and 4.07 for Group F (standard deviation [SD]: 1.45). A required sample size of 35 participants per group was calculated using an online sample size calculator (http://powerandsamplesize.com/Calculators/Compare-2-Means/2-Sample-Non-Inferiority-or-Superiority, accessed on 6 January 2022). Considering a dropout rate of approximately 10%, 40 participants were required in each group, resulting in a total sample size of 120. 

The normality of distribution of continuous variables was examined using the Kolmogorov–Smirnov test. One-way analysis of variance (ANOVA) or Kruskal–Wallis test was used for comparing continuous variables, while Pearson’s χ^2^ test or Fisher’s exact test was used to analyze categorical variables. VAS scores for each group at 24 h after surgery were analyzed using repeated-measures ANOVA, followed by a post hoc test. In the case of significant differences between groups, statistical significance was assessed using *t*-tests with Bonferroni correction. *p*-values < 0.05 were considered statistically significant. Data were presented as means ± SD or frequencies and percentages, as appropriate. All statistical analyses were performed using IBM SPSS Statistics for Windows (v. 26.0; IBM Corp., Armonk, NY, USA).

One-sided non-inferiority testing was performed, comparing the 95% confidence intervals (CIs) of the difference in VAS scores. Non-inferiority was confirmed when the upper 95% CI was lower than the predetermined non-inferiority margin (δ = 1.0).

## 3. Results

**Figure 1:**
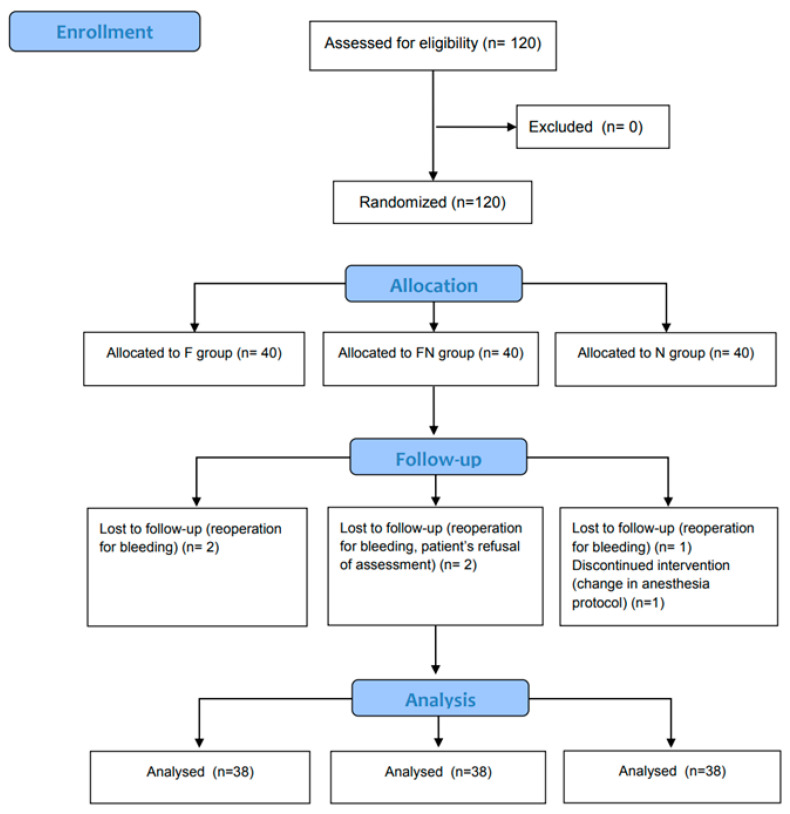
CONSORT flow diagram.

A total of 120 patients were enrolled and randomly allocated to one of the groups, with 114 completing the study (Figure 1).

Analysis of demographic and intraoperative data, including age, body measurements, ASA class, anesthesia time, operation time, total infused anesthetic dose, and reconstruction type or location, showed no significant differences among the three groups (Table 1). The mean PCA doses were as follows: fentanyl 602.9 ± 105.0 mcg in Group F, fentanyl 312.2 ± 68.1 mcg and nefopam 58.7 ± 8.9 mg in Group FN, and nefopam 110.0 ± 9.1 mg in Group N.

The VAS scores in Groups N and FN were not significantly inferior to those in Group F at 24 h after surgery, as the upper and lower limits of the 95% CI fell within the non-inferiority margin (1.0) for both VASr and VASm (Table 2).

**Figure 2:**
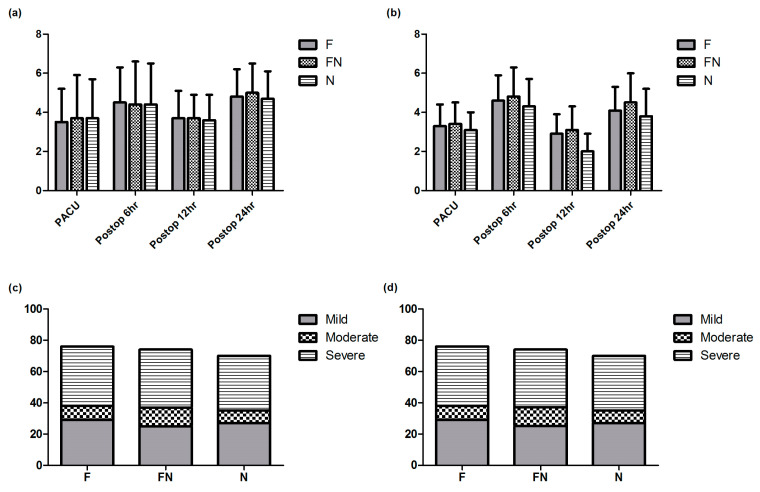
Postoperative pain intensity: (**a**) Postoperative changes in VASr. (**b**) Postoperative changes in VASm. (**c**) VASr intensity level at 24 h after surgery. (**d**) VASm intensity level at 24 h after surgery. VASr: visual analog scale at rest; VASm: visual analog scale during movement.

**Figure 3:**
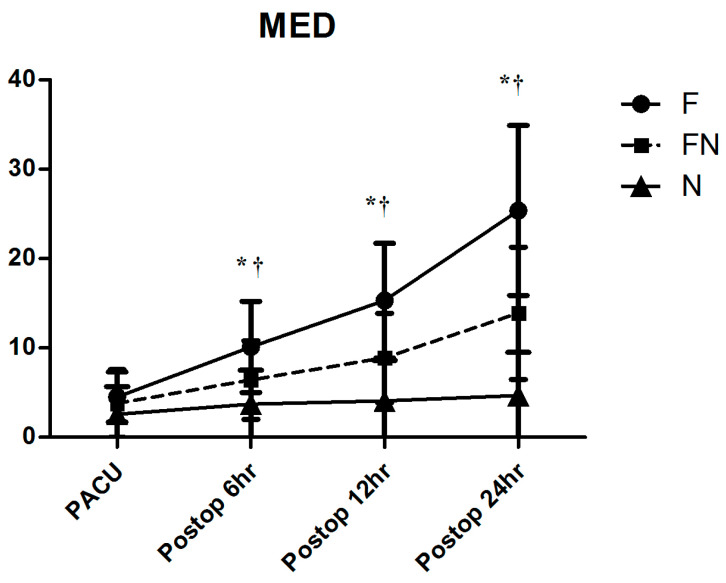
Postoperative cumulative opioid consumption: MED: morphine equivalent dose. *: *p* < 0.05 compared to Group F, †: *p* < 0.05 compared to Group N.

Figure 2 and Figure 3 depict the changes in pain intensity and cumulative postoperative opioid consumption. No significant differences were observed in the VASr and VASm scores, measured in PACU at 6, 12, and 24 h after the surgery, among the three groups. At 24 h postoperatively, the VASr scores were 2.9 ± 1.0, 3.1 ± 1.2, and 2.8 ± 0.9 for Groups F, FN, and N, respectively (*p* = 0.501). Correspondingly, the VASm scores at the same timepoint were 4.1 ± 1.2, 4.5 ± 1.5, and 3.8 ± 1.4, respectively (*p* = 0.129). In addition to the continuous analysis of the VAS score, we analyzed it as a categorical variable. Pain intensity was categorized as follows: 0 indicating no pain, 1–3 indicating mild pain, 4–6 indicating moderate pain, and greater than 6 indicating severe pain. The distribution of categorical VAS scores measured 24 h after the surgery is shown in Figure 2. This categorical analysis revealed no significant differences in the distribution of pain intensity levels among the groups at any timepoint (*p* > 0.05, Table 3), suggesting that the intensity of pain experienced was similar in different analgesic regimes. The MED at 24 h was significantly lower in Group N (25.4 ± 9.5 mg) compared to Group F (13.9 ± 7.4 mg) and Group FN (4.7 ± 4.8 mg). The three groups exhibited significant differences in total opioid consumption at all timepoints except in PACU (*p* < 0.0001). 

There was no clinical difference between the length of PACU stay and the time to initial administration of rescue medication among the groups (Table 3). Furthermore, there was no significant difference between total PCA usage in PACU and within 6 h postoperatively. However, differences among the group became evident over 12 and 24 h postoperatively. The need for rescue medication or the frequency of its administration showed no differences among the groups.

The incidence of PCA-related adverse effects, including PONV, exhibited no significant difference among the three groups (Table 4). Each group had a single instance of PCA discontinuation due to persistent PONV despite antiemetic administration.

## 4. Discussion

This study aimed to assess whether PCA with nefopam was as efficacious as PCA with opioids in patients undergoing immediate BRS after mastectomy. Our findings suggested that PCA with nefopam, either alone or in combination with opioids, was not inferior to PCA with opioids alone in controlling postoperative pain. VAS scores measured at each postoperative timepoint revealed no differences among the three groups. PCA containing nefopam, either alone or in combination with opioids, provided analgesic effects comparable to PCA containing opioids alone, while also reducing opioid consumption. However, there was no significant difference in the incidence of opioid-related complications.

These results were consistent with previous studies, demonstrating the effectiveness of nefopam, whether used as an adjuvant or as a substitute for opioids in PCA, for achieving effective and comparable pain control after surgery [6,7,8,9,10]. Kim et al. reported that nefopam alone provided sufficient analgesic effect following cardiac surgery. Their study found that analgesic efficacy, bolus dose requirements, and total infused PCA volume were similar across PCA regimens utilizing nefopam alone (4 mg/h), fentanyl alone (20 mcg/h), or a combination of fentanyl (10 mcg/h) and nefopam (2 mg/h). Additionally, PCA containing nefopam alone or in combination with fentanyl exhibited comparable analgesic effects with a lower incidence of PONV compared to PCA containing fentanyl alone. Similarly, nefopam-only PCA has demonstrated non-inferior analgesic efficacy compared to nefopam–fentanyl PCA following laparoscopic surgeries. Moreover, adjunctive nefopam administration has been shown to reduce postoperative fentanyl consumption while demonstrating superior analgesic effects after abdominal surgery, including renal transplantation. 

Previous studies have yielded conflicting results regarding PONV incidence when using nefopam as a postoperative analgesic. Some studies have suggested that nefopam itself may not decrease PONV [6,7,8,13] due to its emetic properties [14,15]. However, others have reported that nefopam use could reduce opioid consumption, thereby reducing PONV incidence [9,10]. In our study, both adjunctive therapy and monotherapy with nefopam exhibited opioid-sparing effects of 54% and 18%, respectively, but did not decrease the incidence of PONV. Risk factors associated with PONV, including female sex, non-smoking status, previous history of PONV, and intraoperative opioid use, were prevalent among our participants. However, the overall incidence of PONV in our study was 8.8%, significantly lower than the rates reported in previous studies involving breast surgery patients (60–80%) [16]. This could be attributed to the aggressive use of non-opioid agents with nefopam, prophylactic antiemetic administration, and total intravenous anesthesia (TIVA), especially with propofol, in all study participants. Therefore, despite differences in opioid administration, significant intergroup differences in PONV incidence were not observed.

With increasing emphasis on postoperative recovery, the implementation of enhanced recovery after surgery (ERAS) protocols has become standard practice in surgical settings [16]. An important aspect of anesthesia in ERAS protocols is multimodal analgesic management, aimed at reducing opioid usage while ensuring adequate pain control and minimizing opioid-related complications, such as PONV. Proactive preventive strategies are particularly important for high-risk patients, such as females. In this study, all participants were managed according to our institution’s ERAS protocol, which included reducing opioid usage through multimodal analgesia, administering prophylactic antiemetics, preference for TIVA over inhaled anesthetics, ensuring adequate hydration, and intraoperative temperature regulation. Compared to conventional anesthesia protocols involving inhaled anesthetics and opioid-based analgesia, implementing the ERAS protocol in all study subjects resulted in an approximately 60% reduction in postoperative opioid consumption and a 50% decrease in PONV incidence. Even in Group F, where opioid-based PCA was applied, significant reductions of approximately 33% and 38% were observed, respectively. Additionally, there was a significant decrease of approximately 50% in the requirement for additional analgesics within the first 24 h after surgery. These improvements are expected to enhance patients’ recovery experience and satisfaction levels. Therefore, we advocate for the adoption of this anesthetic management strategy to facilitate optimal recovery in BRS. While our study only adhered to limited aspects of the ERAS protocol, excluding preoperative analgesic medication and nerve blockade in terms of multimodal analgesia [17], it is noteworthy that the quality of patient recovery can be significantly improved by simply implementing TIVA anesthesia, prophylactic antiemetics, and postoperative non-opioid analgesics.

Interestingly, cumulative PCA consumption was higher in Group F compared to Groups N and FN at 12 and 24 h postoperatively. Assuming that the effective doses of fentanyl and nefopam used in all groups were appropriate, this difference may be due to the disparity in the half-lives of fentanyl and nefopam. The half-life of fentanyl is 3–4 h, while that of nefopam is 4–6 h, with nefopam having a slightly longer half-life [18,19]. Therefore, it is presumed that the additional use of PCA bolus was lower in Groups N and FN, resulting in a difference in total consumption.

The side-effects associated with nefopam include nausea, dizziness, headache, dry mouth, sweating, and tachycardia [5,14,20]. Previous studies have indicated that these side-effects are not severe when nefopam is administered at appropriate doses [6,7,9,10]. Neurological complications, such as delirium, confusion, and seizures, are more commonly observed in older patients or those with pre-existing psychological disorders. To ensure that nefopam was administered within the maximum daily dose, the maximum dose was limited to 120 mg when manufacturing the PCA. Patients with advanced age, psychological or neurological disorders, or relevant preoperative medications were excluded from the study. Close monitoring was maintained throughout the study period, and no serious side-effects were observed at the dose used in this investigation.

Pain intensity measurement is a crucial aspect of evaluating postoperative analgesia efficacy. The VAS and the Numerical Rating Scale (NRS) are commonly used tools in clinical practice. The VAS is a continuous scale represented by a 10 cm line where patients mark their pain intensity, making it highly sensitive and capable of detecting small changes in pain levels. However, it can be challenging for some patients to understand and use accurately. On the other hand, the NRS is an 11-point scale where patients rate their pain from 0 (no pain) to 10 (worst pain imaginable). It is preferred by patients due to its simplicity and ease of use. Studies have shown that NRS is often more practical in clinical settings and provides comparable reliability and validity to VAS [21,22].

While NRS is often preferred for its simplicity and patient satisfaction, we utilized the VAS to measure pain intensity both at rest (VASr) and during movement (VASm). This decision was based on our institutional pain management guidelines, which recommend the use of the VAS for its detailed differentiation between static and dynamic pain. The VAS also is a well-validated tool that allows for nuanced differentiation between static and dynamic pain, which is crucial in the postoperative period where activities such as early deep breathing and coughing (EDBC) are essential for recovery. Studies have shown that both VAS and NRS provide reliable and sensitive measures of pain intensity [23,24].

This study had several limitations. Firstly, the precise opioid-equivalent dose of nefopam has not yet been established. We were able to achieve sufficient and non-inferior analgesic effects with an assumed equivalent dose based on similar study results. However, further research is needed to confirm the exact opioid-equivalent dose of nefopam. Secondly, we evaluated the study participants for a relatively short period, as the intravenous line and PCA were removed on the second postoperative day. Lastly, this study was conducted with a limited range of surgical procedures. To generalize the efficacy of nefopam PCA, evidence from various types of surgery with a larger sample size is necessary.

## 5. Conclusions

In conclusion, PCA with nefopam, whether alone or in combination with opioids, provided non-inferior analgesic efficacy compared to PCA with fentanyl alone in patients undergoing immediate BRS following mastectomy. These findings suggest that nefopam may be a valuable alternative or adjunct to opioids in postoperative pain management following BRS.

The English in this document has been checked by at least two professional editors, both native speakers of English. For a certificate, please see http://www.textcheck.com/certificate/9CUfig (accessed on 12 May 2024).

## Figures and Tables

**Table 1:** Patient demographics and intraoperative variables.

Group	F	FN	N	*p*
	(N = 38)	(N = 38)	(N = 38)	
Age (yr)	49.6 ± 7.8	46.9 ± 8.6	46.8 ± 7.7	0.246
Height (cm)	157.8 ± 0.7	160.7 ± 4.4	159.1 ± 5.6	0.069
Weight (kg)	59.0 ± 7.7	61.1 ± 7.5	57.3 ± 5.7	0.076
ASA				0.261
1	19 (50.0%)	18 (47.4%)	24 (63.2%)	
2	19 (50.0%)	20 (52.6%)	13 (34.2%)	
3	0 (0.0%)	0 (0.0%)	1 (2.6%)	
Anesthesia time (min)	288.9 ± 90.7	304.4 ± 115.6	272.4 ± 82.1	0.381
Operation time (min)	248.5 ± 89.2	261.5 ± 109.4	236.5 ± 80.4	0.531
Total infused propofol dose (mg)	2086.8 ± 838.4	2376.9 ± 1094.9	1928.1 ± 658.9	0.096
Total infused remifentanil dose (mcg)	1954.3 ± 546.0	2214.0 ± 882.6	1911.2 ± 639.2	0.143
Type of breast reconstruction				0.417
Implant	30 (78.9%)	27 (71.1%)	26 (68.4%)	
DIEP flap	7 (18.4%)	7 (18.4%)	6 (15.8%)	
LD flap	1 (2.6%)	4 (10.5%)	6 (15.8%)	
Surgical location				0.855
Rt	19 (50.0%)	18 (47.4%)	18 (47.4%)	
Lt	16 (42.1%)	17 (44.7%)	19 (50.0%)	
Both	3 (7.9%)	3 (7.9%)	1 (2.6%)	
PCA	602.9 ± 105.0	312.2 ± 68.1	N/A	
Fentanyl (mcg)				
Nefopam (mg)	N/A	58.7 ± 8.9	110.0 ± 9.1	

Values are expressed as number (proportion) or mean ± standard deviation. DIEP: deep inferior epigastric perforator; LD: latissimus dorsi.

**Table 2:** Non-inferiority test between nefopam and fentanyl in patient-controlled analgesia following breast reconstruction surgery.

	Group F	Group FN	Group N	*p*	Difference between Group F and Group FN	Difference between Group F and Group N
	(N = 38)	(N = 38)	(N = 38)			
VASr 24	2.9 ± 1.0	3.1 ± 1.2	2.8 ± 0.9	0.501	−0.2	−0.1
					(−0.278, 0.678)	(−0.499, 0.299)
VASm 24	4.1 ± 1.2	4.4 ± 1.3	4.1 ± 1.2	0.515	0.3	0
					(−0.184, 0.784)	(−0.478, 0.478)

Values are expressed as mean ± standard deviation or mean (95% confidence intervals). VASr 24: resting visual analogue scale pain score at 24 h after surgery; VASm 24: visual analogue scale pain score with movement 24 h after surgery. Non-inferiority margin defined as 1.0.

**Table 3:** Postoperative recovery.

Group	F	FN	N	*p*
	(N = 38)	(N = 38)	(N = 38)	
Pain intensity level (mild/moderate/severe) VASr				
PACU	18/19/1	17/17/4	18/16/4	0.691
6 h	18/17/3	17/20/1	19/17/2	0.885
12 h	25/12/1	24/14/0	25/13/0	0.968
24 h	29/9/0	26/12/0	27/11/0	0.810
VASm				
PACU	12/21/5	15/16/7	17/12/9	0.356
6 h	8/24/6	7/23/8	8/25/5	0.936
12 h	8/29/1	7/24/7	9/25/4	0.254
24 h	14/23/1	12/24/2	13/23/2	0.988
Length of stay in PACU (min)	47.3 ± 9.7	58.3 ± 5.9	49.8 ± 4.6	0.456
Time to 1st rescue drug administration (min)	8.2 ± 12.6	5.0 ± 8.9	7.1 ± 10.4	0.836
Cumulative PCA consumption (mL)				
PACU	3.6 ± 1.2	2.9 ± 1.2	3.3 ± 0.9	0.026
6 h	13.0 ± 5.4	10.8 ± 4.3	9.4 ± 3.6	0.078
12 h	21.3 ± 7.6	16.5 ± 4.5	15.0 ± 3.8	0.001
24 h	35.9 ± 10.2	31.1 ± 9.4	30.6 ± 7.9	0.026
Rescue analgesic requirement (Yes)				
PACU	18 (47.4%)	16 (42.1%)	18 (47.4%)	0.540
24 h	13 (34.2%)	15 (39.5%)	13 (34.2%)	0.911
No. of rescue drugs administered (1st/2nd/3rd)				
PACU	17/1/0	13/3/0	16/2/0	0.797
24 h	11/ 1/1	12/3/0	10/2/1	0.929

Values are expressed as number (proportion) or mean ± standard deviation. VASr: visual analog scale at rest; VASm: visual analog scale during movement.

**Table 4:** PCA-related postoperative complications.

Group	F	FN	N	*p*
	(N = 38)	(N = 38)	(N = 38)	
Side effects (Y)				
PONV	4 (10.5%)	5 (13.2%)	1 (2.6%)	0.337
Dizziness	5 (13.2%)	4 (10.5%)	2 (5.3%)	0.619
Ileus	3 (7.9%)	1 (2.6%)	1 (2.6%)	0.616
Urinary retention	1 (2.6%)	1 (2.6%)	1 (2.6%)	1.000
Respiratory depression	0 (0.0%)	0 (0.0%)	0 (0.0%)	1.000
Hypotension	0 (0.0%)	0 (0.0%)	0 (0.0%)	1.000
PCA withdrawal d/t PONV	1 (2.6%)	1 (2.6%)	1 (2.6%)	1.000

Values are expressed as number (proportion) or mean ± standard deviation.

## Data Availability

The data generated in this study can be shared after a reasonable request to the corresponding author.
